# ^18^F‐FGln PET/CT Imaging of Glutamine Uptake Has Potential for Predicting Immunotherapy Response in Hepatocellular Carcinoma

**DOI:** 10.1002/advs.78622

**Published:** 2026-11-03

**Authors:** Yi Liu, Jindian Li, Xiaoyu Pan, Xiaoping Xu, Xuan Huang, Simin He, Jianping Zhang, Fei Liu, Shaoli Song

**Affiliations:** ^1^ Department of Nuclear Medicine Fudan University Shanghai Cancer Center Shanghai China; ^2^ Department of Nuclear Medicine Clinical Oncology School of Fujian Medical University Fujian Cancer Hospital Fuzhou China; ^3^ Department of Oncology Shanghai Medical College Fudan University Shanghai China; ^4^ Center For Biomedical Imaging Fudan University Shanghai China; ^5^ Shanghai Engineering Research Center of Molecular Imaging Probes Shanghai China

**Keywords:** ^18^F‐FGln PET/CT, glutamine uptake, hepatocellular carcinoma, immunotherapy

## Abstract

We investigated whether glutamine (Gln) uptake, imaged by ^18^F‐(2S,4R)‐4‐fluoroglutamine (^18^F‐FGln) PET/CT, could predict and modulate immunotherapy response in hepatocellular carcinoma (HCC). Analysis of the Cancer Genome Atlas (TCGA)‐Liver Hepatocellular Carcinoma (LIHC) dataset revealed that *SLC1A5* expression—which encodes a primary Gln uptake channel in HCC cells—was significantly associated with poor prognosis, higher tumor grade, and an immunosuppressive tumor microenvironment. In murine HCC models, tumors with high ^18^F‐FGln uptake exhibited elevated *Slc1a5* expression and poor response to anti‐PD‐1 therapy, whereas low‐uptake tumors were more sensitive to immunotherapy. Genetic knockdown of *Slc1a5* reduced ^18^F‐FGln uptake, enhanced anti‐PD‐1 efficacy, increased cytotoxic T lymphocyte infiltration, and decreased regulatory T cell abundance. Transcriptomic and single‐cell analyses revealed activation of antitumor immune pathways and reprogramming toward an immune‐inflamed phenotype after *Slc1a5* suppression. V‐9302 decreased tumor Gln uptake, as measured by ^18^F‐FGln PET/CT, and sensitized HCC to PD‐1 blockade. In patient‐derived organoid‐T cell co‐culture systems, low‐SLC1A5 organoids were susceptible to PD‐1 blockade, whereas high‐SLC1A5 organoids were resistant, consistent with ^18^F‐FGln PET/CT findings. These results identify Gln uptake as a checkpoint of immune evasion in HCC and support that ^18^F‐FGln PET/CT has the potential to predict and monitor immunotherapy response.

## Graphical abstract

^18^F‐FGln PET/CT imaging serves as a marker of Gln uptake in hepatocellular carcinoma. Tumors with high Gln uptake drive Treg‐mediated immunosuppression and immune evasion during antitumor immunotherapy. Conversely, tumors with low Gln uptake maintain sufficient Gln in the TME, suppressing Tregs and enabling cytotoxic T cells to inhibit tumor growth under antitumor immunotherapy.

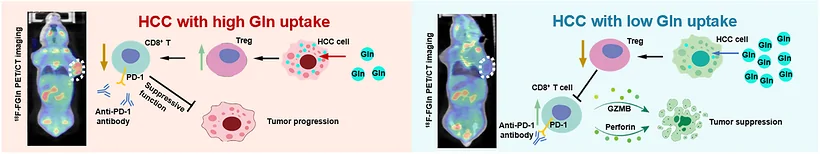

## 1 Introduction

Hepatocellular carcinoma (HCC) remains a major global health burden and ranks as the third leading cause of cancer‐related death worldwide. Its incidence and mortality are projected to increase by more than 50% by 2040 [1]. Although systemic therapies have advanced substantially, objective response rates in real‐world clinical practice remain limited, at approximately 20% [2]. The introduction of immunotherapies, particularly immune checkpoint inhibitors (ICIs), has transformed the therapeutic landscape of HCC [3]. However, durable clinical benefit remains confined to a subset of patients because of primary and acquired resistance. For example, in the phase III LEAP‐002 trial, lenvatinib plus pembrolizumab prolonged median overall survival compared with lenvatinib monotherapy (21.2 vs. 19.0 months), but did not meet the prespecified threshold for statistical significance [4]. These findings highlight a critical clinical challenge: a substantial proportion of patients are refractory to, or fail to derive durable benefit from, immunotherapeutic interventions.

Metabolic reprogramming within the tumor microenvironment (TME) is increasingly recognized as a key driver of tumor progression and immune evasion. Among these metabolic alterations, glutamine (Gln) metabolism plays a critical role in sustaining tumor growth and promoting immunosuppressive signaling [5]. As a major anaplerotic substrate, Gln supports biosynthesis and redox homeostasis in rapidly proliferating cancer cells while also regulating immune cell differentiation and function [6]. Importantly, competition for Gln between tumor cells and immune cells can impair T‐cell function and weaken antitumor immunity [7]. Consistently, Gln deprivation suppresses T‐lymphocyte proliferation and cytokine production [8], underscoring the central role of Gln metabolism in shaping antitumor immune responses. Together, these observations suggest that Gln metabolic reprogramming may be an important mechanism underlying resistance to immunotherapy in HCC.

Emerging evidence further suggests that targeting Gln metabolism may enhance immunotherapeutic efficacy by remodeling the TME. The Gln transporter solute carrier family 1 member 5 (SLC1A5; also known as ASCT2) is frequently upregulated in cancer cells and serves as a major route of Gln uptake. Pharmacological inhibition of SLC1A5 with the small‐molecule inhibitor V‐9302 selectively reduces Gln uptake in tumor cells while sparing CD8^+^ T cells, thereby enhancing T‐cell effector function [9]. In multiple tumor models, combining Gln‐targeted interventions with ICIs has demonstrated synergistic antitumor activity and improved immune remodeling of the TME [10, 11]. These findings support the rationale for targeting Gln metabolism to overcome immunotherapy resistance. However, the metabolic heterogeneity of HCC and the lack of noninvasive approaches for assessing tumor Gln utilization remain major barriers to clinical translation.

Tumor metabolic heterogeneity is a defining feature of cancer and has important therapeutic implications. Distinct metabolic phenotypes may confer differential sensitivity to targeted therapies, underscoring the need for patient stratification based on tumor metabolic features. In this context, noninvasive imaging approaches capable of quantifying tumor‐specific metabolic pathways are of particular clinical value. The clinical success of 2‐deoxy‐2‐[^18^F] fluoro‐D‐glucose (^18^F‐FDG) PET/CT, which exploits increased glucose uptake in tumors, illustrates the value of metabolic imaging. Extending this paradigm, ^18^F‐(2S,4R)‐4‐fluoroglutamine (^18^F‐FGln) has been developed as a PET tracer for interrogating Gln uptake in vivo [12]. Mechanistically, ^18^F‐FGln is transported by both sodium‐independent system L and sodium‐dependent system ASC transporters, with preferential uptake mediated by SLC1A5 [13]. Importantly, ^18^F‐FGln PET enables noninvasive assessment of tumor Gln utilization and can capture pharmacodynamic responses to therapies targeting this pathway [12].

In the present study, we investigated the utility of ^18^F‐FGln PET/CT for predicting and monitoring responses to combined SLC1A5 inhibition and immunotherapy in preclinical HCC models. We show that tumors with low ^18^F‐FGln uptake are sensitive to anti‐PD‐1 therapy, whereas those with high uptake exhibit resistance. V‐9302 effectively suppresses tumor Gln uptake and sensitizes resistant tumors to anti‐PD‐1 treatment. In organoid‐T‐cell co‐culture experiments, SLC1A5‐low organoids were markedly suppressed by immunotherapy, whereas SLC1A5‐high organoids were resistant. Collectively, our findings support the translational potential of ^18^F‐FGln PET/CT as a noninvasive strategy for predicting and monitoring HCC responses to immunotherapy and for guiding precision Gln uptake inhibition. This study provides a rationale for future clinical trials using ^18^F‐FGln PET/CT to stratify and monitor patients with HCC receiving combined Gln uptake inhibition and ICI therapy.

## 2 Results

### 2.1 The Expression of *SLC1A5* in HCC Is Associated with Poorer Prognosis and an Immune‐Desert Phenotype

Gln has attracted considerable attention because of its metabolic roles in cancer cells [14, 15]. Recent studies have revealed metabolic crosstalk involving Gln between tumor cells and immune cells, which shapes antitumor immunity and influences tumor progression [16]. Elevated expression of *SLC1A5*, a major Gln transporter, in cancer cells suppresses the antitumor function of cytotoxic CD8^+^ T cells within the TME [5]. Analysis of The Cancer Genome Atlas (TCGA)‐Liver Hepatocellular Carcinoma (LIHC) dataset showed that higher *SLC1A5* expression in HCC was associated with poorer prognosis (Figure 1A). Kaplan‐Meier survival analysis showed that patients with high *SLC1A5* expression had significantly lower overall survival than those with low *SLC1A5* expression (log‐rank test, *p* < 0.001).

**FIGURE 1:**
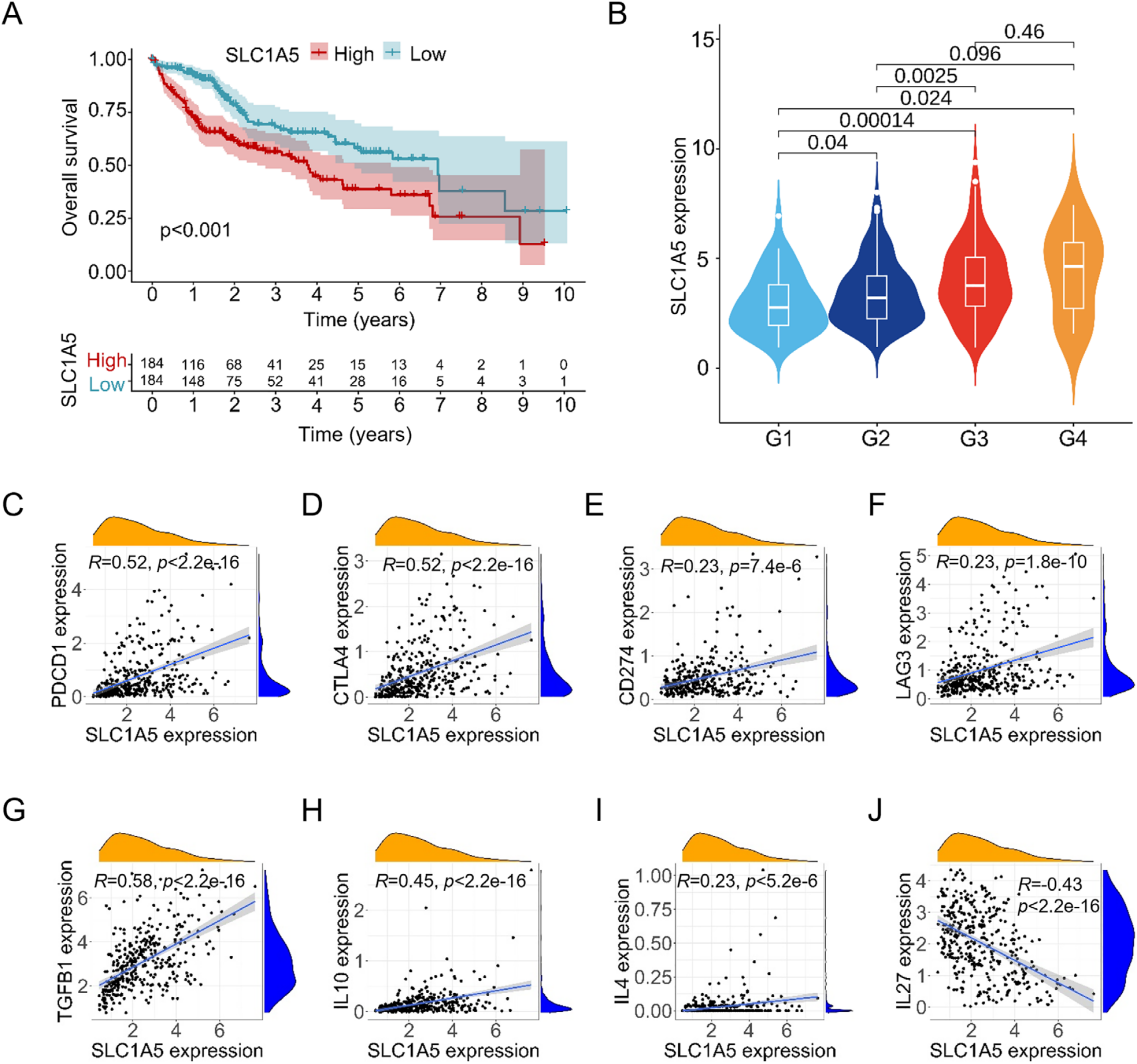
*SLC1A5* expression is associated with poor prognosis and immune‐desert features in HCC. (A) Kaplan‐Meier analysis of overall survival in patients with HCC stratified by *SLC1A5* expression using the TCGA‐LIHC dataset. (B) Association between *SLC1A5* expression and histological grade in patients with HCC from the TCGA‐LIHC dataset. (C–J) Correlations between *SLC1A5* expression and the expression of *PDCD1*, *CTLA4*, *CD274*, *LAG3*, *TGFB1*, *IL10*, *IL4*, and *IL27* in patients with HCC from the TCGA‐LIHC dataset.

Histological grade, which reflects the degree of tumor malignancy, was correlated with *SLC1A5* expression in HCC. Higher *SLC1A5* expression was associated with higher histological grade and greater tumor malignancy (Figure 1B). Moreover, *SLC1A5* expression was positively correlated with the expression of immune checkpoint genes, including *PDCD1*, *CTLA4*, *CD274*, and *LAG3* (Figure 1C–F). *SLC1A5* expression was also positively correlated with immunosuppressive cytokines, including *TGF‐β, IL‐10, and IL‐4* (Figure 1G–I). Conversely, *SLC1A5* expression was negatively correlated with *IL‐27*, an immune‐enhancing cytokine (Figure 1J). These findings suggest that *SLC1A5* expression in HCC is associated with the formation of an immunosuppressive TME and poorer prognosis.

### 2.2 High ^18^F‐FGln Uptake on PET/CT Predicts Poor Response to Immunotherapy in HCC

SLC1A5 is a plasma membrane transporter responsible for Gln uptake and has been identified as a critical mediator of growth and survival in multiple cancer types [17]. Many cancer cells undergo Gln‐centered metabolic reprogramming, increasing Gln uptake to support rapid proliferation [18]. In rapidly dividing immune cells, such as lymphocytes, Gln consumption is comparable to or even exceeds glucose consumption [19]. Recent studies have shown that Gln uptake within the TME is predominantly driven by cancer cells [20]. Gln deficiency in the TME markedly impairs T‐cell activation, thereby compromising the efficacy of immunotherapy [21]. The PET radiotracer ^18^F‐FGln is transported by the same cellular transporters as native Gln but undergoes minimal metabolism [22]. Thus, ^18^F‐FGln has been developed as an in vivo imaging probe for assessing tumor Gln uptake [23]. Therefore, we hypothesized that ^18^F‐FGln PET/CT imaging could be used to identify patients who are more likely to benefit from immunotherapy.

We first evaluated the Gln uptake capacity of several HCC cell lines, including CBRH‐7919, H22, Hepa1‐6, RH‐35, and Hepa1c1c7, using cellular uptake assays (Figure S1A). On the basis of these results, H22 cells, characterized by high Gln uptake, and Hepa1‐6 cells, characterized by low Gln uptake, were selected to establish tumor models for ^18^F‐FGln micro‐PET/CT imaging. H22 tumors exhibited high ^18^F‐FGln uptake, whereas Hepa1‐6 tumors showed low uptake (Figure 2A). Quantitative analysis revealed that ^18^F‐FGln uptake was significantly higher in H22 tumors than in Hepa1‐6 tumors (26.30 ± 2.30%ID/g vs. 6.87 ± 0.21%ID/g, *p* < 0.05; Figure 2B). The tumor‐to‐normal tissue (T/NT) ratios were also higher in H22 tumors than in Hepa1‐6 tumors (7.39 ± 0.93 vs. 1.50 ± 0.15, *p* < 0.05; Figure 2C). Autoradiography and H&E staining further confirmed high ^18^F‐FGln accumulation in H22 tumors and low accumulation in Hepa1‐6 tumors (Figure 2D,E). Biodistribution analysis showed that ^18^F‐FGln uptake was 14.04 ± 0.91%ID/g in H22 tumor tissues and 5.31 ± 0.66%ID/g in Hepa1‐6 tumor tissues (*p* < 0.05; Figure S1B).

**FIGURE 2:**
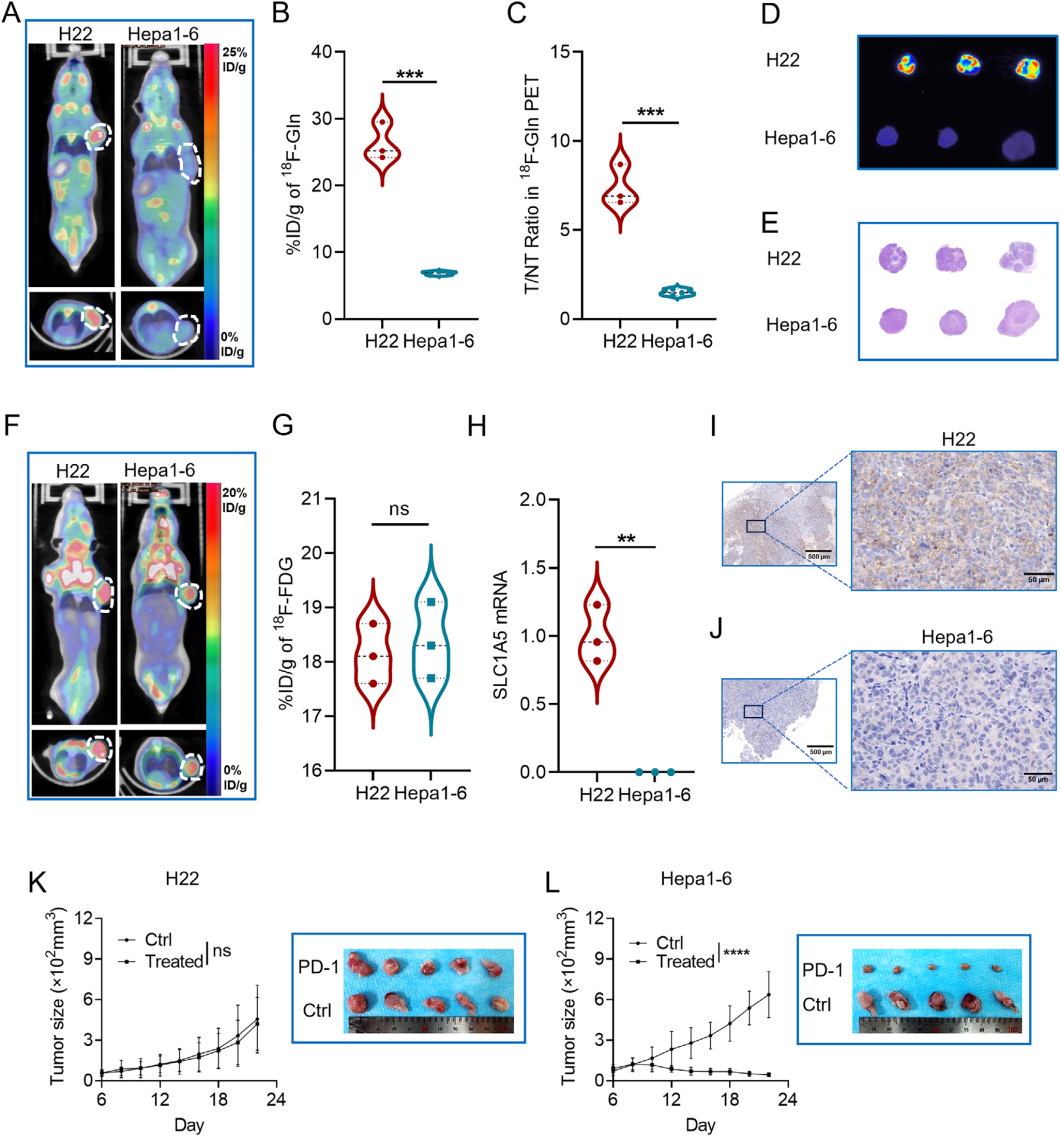
High ^18^F‐FGln uptake is associated with poor response to immunotherapy in HCC. (A) Representative ^18^F‐FGln micro‐PET/CT images of H22 and Hepa1‐6 tumor models. White dashed circles indicate tumor locations. (B) Quantification of tumoral ^18^F‐FGln uptake in H22 and Hepa1‐6 tumor models (n = 3; ***p = 0.0003). (C) Tumor‐to‐normal tissue (T/NT) ratios derived from ^18^F‐FGln micro‐PET/CT images of H22 and Hepa1‐6 tumor models (n = 3; ***p = 0.0009). (D) Representative autoradiography images of H22 and Hepa1‐6 tumors. (E) Representative H&E‐stained sections of H22 and Hepa1‐6 tumors corresponding to autoradiography images. (F) Representative ^18^F‐FDG micro‐PET/CT images of H22 and Hepa1‐6 tumor models. White dashed circles indicate tumor locations. (G) Quantification of tumoral ^18^F‐FDG uptake in H22 and Hepa1‐6 tumor models (n = 3; ns, *p* = 0.6742). (H) Relative *Slc1a5* mRNA expression in H22 and Hepa1‐6 tumor tissues determined by qRT‐PCR (n = 3; **p = 0.0012). (I) Representative IHC staining of SLC1A5 in H22 tumor tissues. Scale bars: 500 µm and 50 µm. (J) Representative IHC staining of SLC1A5 in Hepa1‐6 tumor tissues. Scale bars: 500 µm and 50 µm. (K) Tumor growth curves and representative photographs of H22 tumors after treatment with anti‐mouse PD‐1 antibody (10 mg/kg) or saline (n = 5; ns, *p* = 0.6181). (L) Tumor growth curves and representative photographs of Hepa1‐6 tumors after treatment with anti‐mouse PD‐1 antibody (10 mg/kg) or saline (n = 5; ****p < 0.0001).

To compare the performance of ^18^F‐FGln PET/CT with the widely used clinical standard ^18^F‐FDG PET/CT for monitoring immunotherapy responses, we performed ^18^F‐FDG micro‐PET/CT imaging on both tumor models following the completion of ^18^F‐FGln scans. Notably, both H22 and Hepa1‐6 tumor models exhibited high ^18^F‐FDG uptake (Figure 2F). Quantitative analysis showed comparable ^18^F‐FDG uptake between H22 and Hepa1‐6 tumors (18.13 ± 0.45%ID/g vs. 18.37 ± 0.57%ID/g, *p* > 0.05; Figure 2G). The T/NT ratios were 2.62 ± 0.65 for H22 tumors and 2.71 ± 0.12 for Hepa1‐6 tumors (*p* > 0.05; Figure S1C).

Quantitative reverse transcription polymerase chain reaction (qRT‐PCR) and immunohistochemistry (IHC) confirmed high SLC1A5 expression in H22 tumor tissues and low SLC1A5 expression in Hepa1‐6 tumor tissues (Figure 2H–J). Subsequent evaluation of anti‐PD‐1 therapy showed that PD‐1 blockade significantly suppressed Hepa1‐6 tumor growth but had minimal effects on H22 tumors (Figure 2K,L and Figure S1D,F). Body weight monitoring showed no significant differences between the anti‐PD‐1‐treated and control groups in either tumor model, suggesting an acceptable safety profile (Figure S1E,G). Collectively, these results indicate that tumors with high *SLC1A5* expression exhibit high ^18^F‐FGln uptake and poor responsiveness to anti‐PD‐1 therapy, whereas tumors with low *SLC1A5* expression show low ^18^F‐FGln uptake and marked sensitivity to PD‐1 blockade.

### 2.3 *Slc1a5* Knockdown Reduces ^18^F‐FGln Uptake and Sensitizes HCC to Immunotherapy

To further determine whether *Slc1a5* expression in HCC cells limits the efficacy of immunotherapy, we established an H22 cell line with stable *Slc1a5* knockdown, as confirmed by qRT‐PCR and IHC (Figure 3A–C). H22 KD cells showed lower ^18^F‐FGln uptake than H22 WT (Figure S2A). Tumor models were then generated using H22 cells transduced with a scramble shRNA control vector (H22 WT) and *Slc1a5*‐knockdown H22 cells (H22 KD), and the effect of anti‐PD‐1 therapy on tumor growth was evaluated. Compared with H22 WT tumors, H22 KD tumors were significantly more responsive to PD‐1 blockade, as reflected by greater suppression of tumor growth (Figure 3D,E).

**FIGURE 3:**
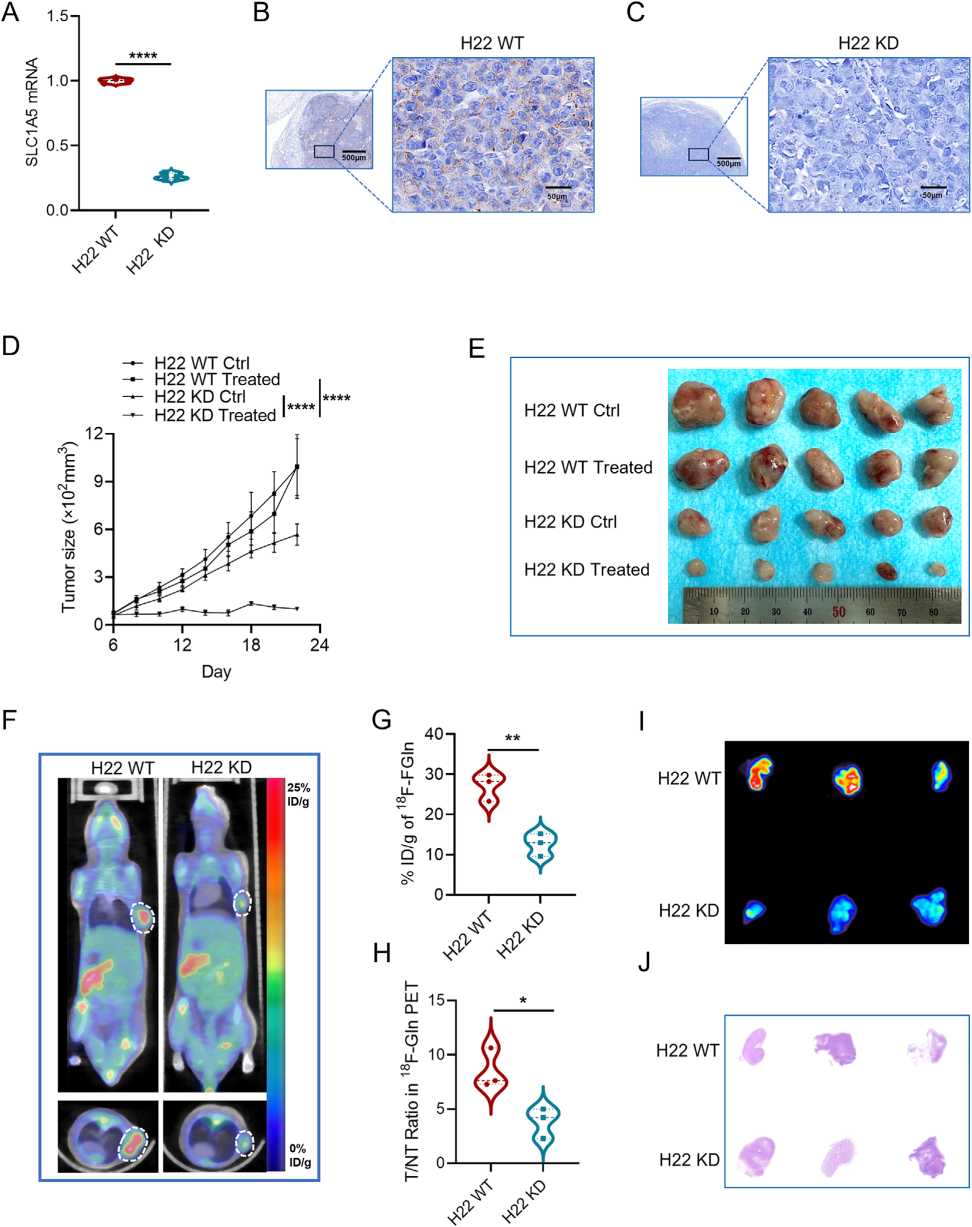
*Slc1a5* knockdown reduces ^18^F‐FGln uptake and sensitizes HCC to immunotherapy. (A) Relative *Slc1a5* mRNA expression in H22 *Slc1a5*‐WT and *Slc1a5*‐KD tumor tissues determined by qRT‐PCR (n = 3; ****p < 0.0001). (B and C) Representative IHC staining of SLC1A5 in H22 *Slc1a5*‐WT and *Slc1a5*‐KD tumor tissues. Scale bars: 500 and 50 µm. (D) Tumor growth curves of H22 *Slc1a5*‐WT and H22 *Slc1a5*‐KD tumors after treatment with anti‐mouse PD‐1 antibody (10 mg/kg) or saline (n = 5; ****p < 0.0001). (E) Representative photographs of H22 *Slc1a5*‐WT and H22 *Slc1a5*‐KD tumors after treatment with anti‐mouse PD‐1 antibody or saline. (F) Representative ^18^F‐FGln micro‐PET/CT images of H22 *Slc1a5*‐WT and H22 *Slc1a5*‐KD tumor models. White dashed circles indicate tumor locations. (G) Quantification of tumoral ^18^F‐FGln uptake in H22 WT and H22 KD tumor models (n = 3; **p = 0.0047). (H) Tumor‐to‐normal tissue (T/NT) ratios derived from ^18^F‐FGln micro‐PET/CT images of H22 WT and H22 KD tumor models (n = 3; *p = 0.0252). (I) Representative autoradiography images of H22 WT and H22 KD tumors. (J) Representative H&E‐stained sections of H22 WT and H22 KD tumors corresponding to autoradiography images.

To further assess whether ^18^F‐FGln PET/CT imaging can guide immunotherapy, we performed micro‐PET/CT scans in both H22 WT and H22 KD tumor models. ^18^F‐FGln uptake was significantly higher in H22 WT tumors than in H22 KD tumors (Figure 3F). Quantitative analysis showed maximum uptake values of 27.10 ± 2.77%ID/g in H22 WT tumors and 12.60 ± 2.30%ID/g in H22 KD tumors (*p* < 0.05; Figure 3G). The T/NT ratios were also significantly higher in H22 WT tumors than in H22 KD tumors (8.51 ± 1.51 vs. 3.83 ± 1.14, *p* < 0.05; Figure 3H). Autoradiography and H&E staining further confirmed high ^18^F‐FGln accumulation in H22 WT tumors and reduced accumulation in H22 KD tumors (Figure 3I,J). Biodistribution analysis revealed significantly higher ^18^F‐FGln uptake in H22 WT tumor tissues than in H22 KD tumor tissues (16.26 ± 1.41%ID/g vs. 5.22 ± 0.48%ID/g, *p* < 0.05; Figure S2B).

Collectively, these results demonstrate that *Slc1a5* knockdown enhances the efficacy of anti‐PD‐1 immunotherapy and that ^18^F‐FGln PET/CT imaging may be useful for monitoring immunotherapeutic responses in HCC.

### 2.4 HCC Intrinsic *Slc1a5* Deficiency Triggers Antitumor Immunity

To investigate the mechanism by which *Slc1a5* knockdown enhances tumor immunotherapy, we performed transcriptomic profiling of H22 KD and H22 WT tumor tissues from BALB/c mice. A total of 1,430 differentially expressed genes (DEGs) were identified, including 1,343 upregulated genes and 87 downregulated genes (Figure 4A). Intersecting the DEGs with a curated immune‐related gene set identified 328 upregulated and 8 downregulated immune‐related genes in H22 KD tumors (Figure S3A). Notably, genes encoding cytotoxic effector molecules, including *Cd8b1*, *Cd8a*, *Prf1*, *Gzmb*, and *Gzmf*, were markedly upregulated (Figure 4A). These transcriptional changes were validated by qRT‐PCR, which confirmed increased mRNA expression of *Cd8b1*, *Cd8a*, *Prf1*, *Gzmb*, and *Gzmf* in H22 KD tumors relative to H22 WT controls (Figure 4B).

**FIGURE 4:**
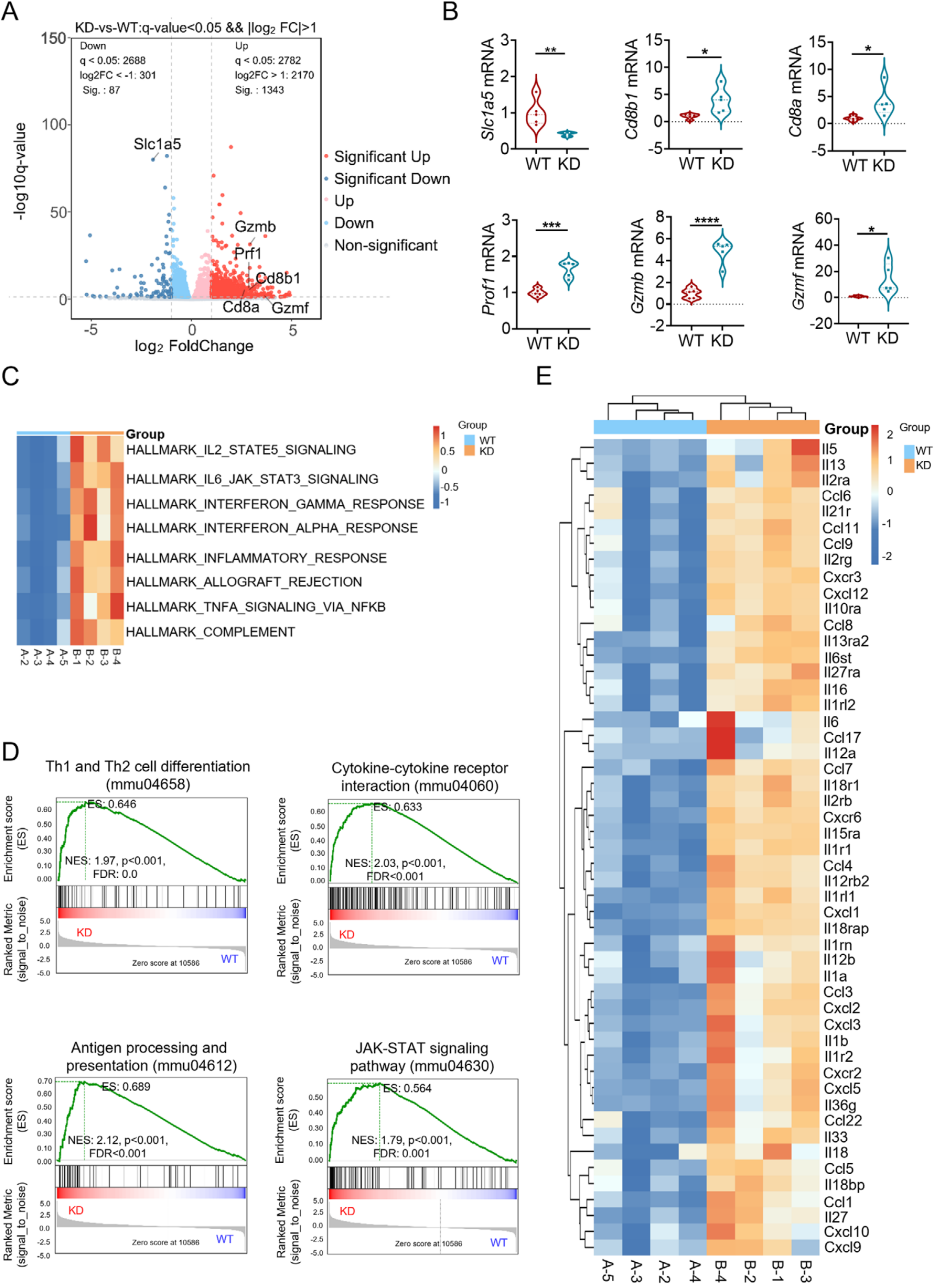
HCC intrinsic *Slc1a5* deficiency triggers antitumor immunity. (A) Volcano plot of RNA‐seq data comparing H22 KD and H22 WT tumors inoculated into BALB/c mice (n = 4 biologically independent samples per group). Differentially expressed genes were identified using thresholds of |log2(fold change)| > 1 and false discovery rate (FDR) < 0.05. (B) Relative Slc1a5, Cd8b1, Cd8a, Prf1, Gzmb, and Gzmf mRNA expression in H22 KD and H22 WT tumors determined by qRT‐PCR (n = 5; *p < 0.05). (C) Hallmark pathway activation profiles showing significant upregulation of immune‐related pathways in the H22 KD group compared with the H22 WT group. (D) Gene set enrichment analysis (GSEA) of H22 KD and H22 WT tumors (n = 4 biologically independent samples per group). (E) Heatmap of RNA‐seq data showing expression of interleukin and chemokine family members.

To characterize the broader signaling networks affected by *Slc1a5* loss, we performed gene set variation analysis (GSVA) using Hallmark and KEGG pathway collections. This analysis showed that *Slc1a5*‐KD tumors exhibited significantly higher enrichment scores for multiple inflammatory and immune‐related pathways, including IL2‐STAT5 signaling and interferon‐gamma response in the Hallmark collection (Figure 4C), as well as Th1/Th2 cell differentiation and antigen processing and presentation in the KEGG collection (Figure S3B). Gene set enrichment analysis (GSEA) further showed that multiple pathways involved in antitumor immunity were significantly enriched in H22 KD tumors, including Th1/Th2 cell differentiation (mmu04658), cytokine‐cytokine receptor interaction (mmu04060), antigen processing and presentation (mmu04612), and JAK‐STAT signaling (mmu04630) (Figure 4D). Consistent with these pathway‐level changes, analysis of RNA‐seq data showed that most members of the interleukin and chemokine families were upregulated in H22 KD tumors (Figure 4E). Collectively, these findings indicate that *Slc1a5* knockdown reshapes the tumor immune microenvironment toward an immune‐inflamed phenotype and may promote antitumor immune responses.

### 2.5 *Slc1a5* Knockdown Shifts the Tumor Immune Microenvironment from Suppression to Activation through Treg Reduction

To characterize immune microenvironmental heterogeneity at single‐cell resolution, we performed single‐cell transcriptomic analysis of tumor‐infiltrating CD45^+^ immune cells isolated from H22 KD and H22 WT tumors. Tumor tissues from three subcutaneous tumors were pooled into a single sample for single‐cell RNA sequencing. Uniform Manifold Approximation and Projection (UMAP) was used to visualize cellular heterogeneity and identify distinct cell clusters in H22 WT and H22 KD tumors. Fourteen cell populations were identified, including CD4^+^ T cells, CD8^+^ T cells, γδ (gd) T cells, natural killer (NK) cells, B cells, plasma cells, monocytes, macrophages, dendritic cells (DCs), neutrophils, mast cells, endothelial cells, and fibroblast‐like cells (Figure 5A). Overall, *Slc1a5* knockdown increased both CD4^+^ and CD8^+^ T‐cell infiltration within tumor tissues (Figure 5B). Given the central role of CD4^+^ T cells in orchestrating the tumor immune microenvironment, we next performed subset‐level analysis of CD4^+^ T cells, which were classified into five distinct subsets: naïve CD4^+^ T cells, proliferating CD4^+^ T cells, central memory CD4^+^ T cells, tissue‐resident memory CD4^+^ T cells, and regulatory T (Treg) cells (Figure 5C). Notably, *Slc1a5* knockdown substantially reduced the proportion of Treg cells (Figure 5D). We further characterized the expression profiles of immunosuppression‐related genes across CD4^+^ T‐cell subsets. Treg cells exhibited significantly elevated expression of multiple immune‐inhibitory genes, including *Foxp3*, *Ctla4*, *Il10*, *Tnfrsf4*, *Tnfrsf9*, *Tnfrsf1b*, *Il2ra*, *Il2rb*, *Icos*, *Ccr2*, *Ccr5*, *Prdm1*, *Klrg1*, *Cd27*, and *Entpd1* (Figure S4A,B).

**FIGURE 5:**
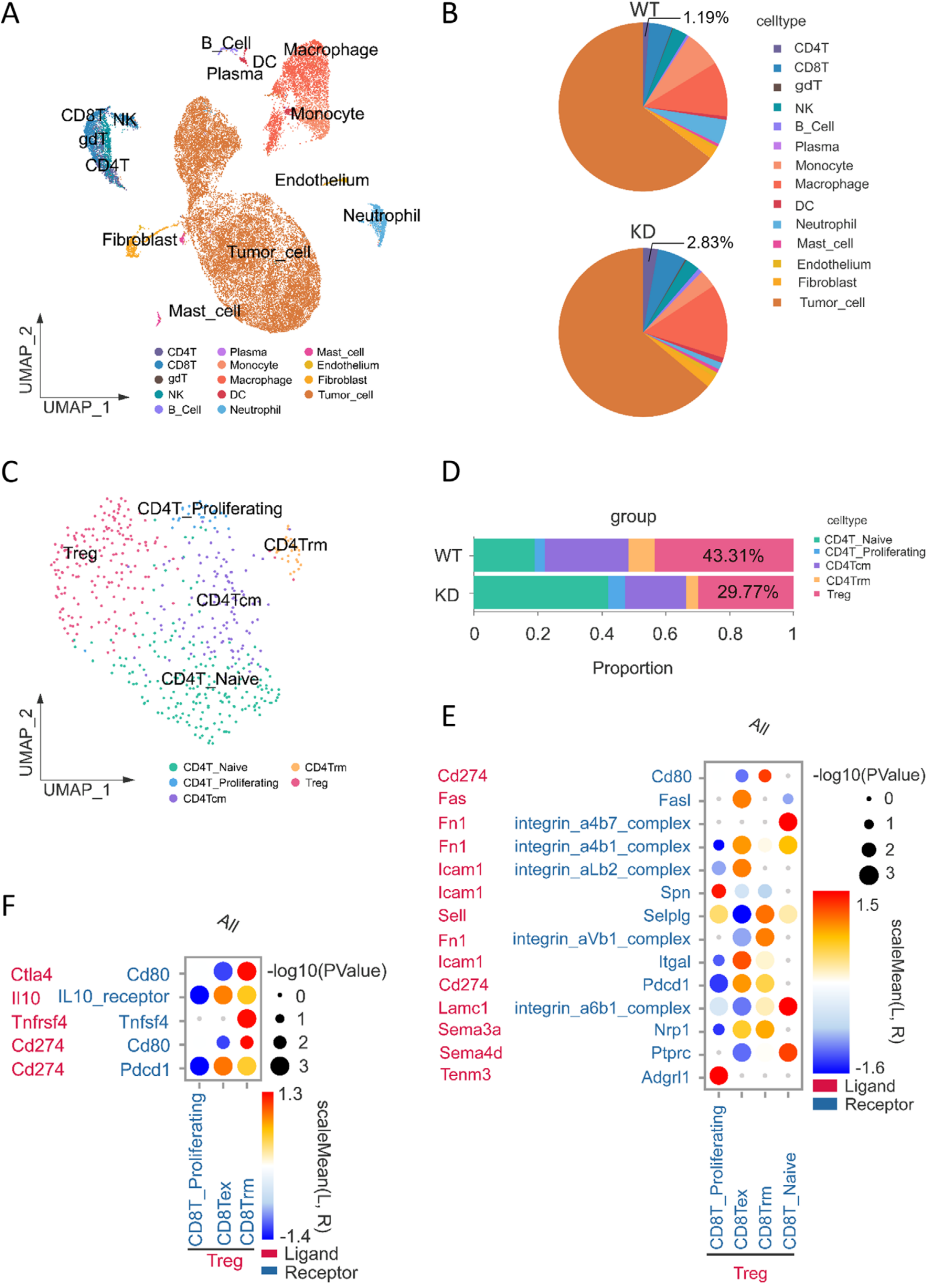
Single‐cell RNA‐seq analysis reveals distinct immune landscapes in *Slc1a5*‐high and *Slc1a5*‐low HCC. (A) UMAP plot of single‐cell transcriptomes colored according to major cell‐type annotations. (B) Pie charts showing the overall cell‐type composition of H22 WT and H22 KD samples. (C) UMAP embedding of re‐clustered CD4^+^ T cells. (D) Relative abundance of CD4^+^ T‐cell subsets in H22 WT and H22 KD samples, shown as stacked bar plots. (E) Bubble plot of cell‐cell communication events derived from intersecting ligand genes with differentially expressed genes. Ligands and receptors are shown according to interacting cell populations; dot size represents significance calculated using CellPhoneDB, and color indicates scaled mean interaction strength or expression. (F) Bubble plot of Treg‐derived immunoregulatory ligand‐receptor interactions with CD8^+^ T‐cell subsets.

To investigate intercellular crosstalk between Treg cells and CD8^+^ T‐cell subsets within the TME, we performed ligand‐receptor interaction analysis. The heatmap in Figure 5E shows the ligand‐receptor pairs identified between interacting cell populations. Notably, interactions involving *Ctla4*, *Il10*, *Tnfrsf4*, and *Cd274* and their cognate receptors were predominantly detected in recipient CD8^+^ T‐cell subsets, including proliferating CD8^+^ T cells (CD8T_Proliferating), exhausted CD8^+^ T cells (CD8T_Tex), and tissue‐resident memory CD8^+^ T cells (CD8T_Trm) (Figure 5F). These findings suggest that SLC1A5‐associated remodeling of ligand‐receptor communication may contribute to resistance to PD‐1 blockade in SLC1A5‐high tumors.

To further validate the conclusions drawn from the single‐cell transcriptomic analysis, we next performed flow cytometry to compare the proportions of Treg cells and cytotoxic T lymphocytes (CTLs) between *Slc1a5*‐KD and *Slc1a5*‐WT tumor tissues. Notably, *Slc1a5* knockdown markedly reduced the proportion of CD25^+^FOXP3^+^ Treg cells, from 11.68 ± 5.45% in the H22 WT group to 2.33 ± 1.80% in the H22 KD group (*p* < 0.05; Figure 6A). In parallel, *Slc1a5* knockdown significantly increased CTL frequencies, as shown by an increase in CD8^+^GZMB^+^ cells from 5.91 ± 1.07% to 12.50 ± 2.03% (*p* < 0.05; Figure 6B) and CD8^+^Perforin^+^ cells from 1.63 ± 0.85% to 6.54 ± 1.56% (*p* < 0.05; Figure 6C).

**FIGURE 6:**
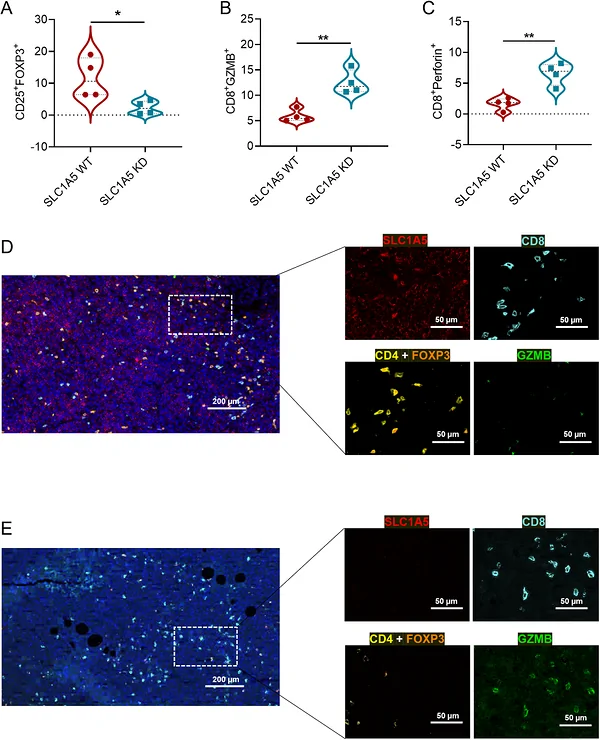
*Slc1a5* knockdown reprograms the TME through Treg reduction. (A‐C) Proportions of CD25^+^FOXP3^+^ T cells, CD8^+^GZMB^+^ T cells, and CD8^+^Perforin^+^ T cells in H22 KD and H22 WT tumors, as measured by flow cytometry (n = 4 biologically independent samples per group). (D) Multicolor immunofluorescence staining of SLC1A5 (red), CD8 (cyan), CD4 (yellow), FOXP3 (orange), and GZMB (green) in H22 SLC1A5‐WT tumor tissues. (E) Multicolor immunofluorescence staining of SLC1A5 (red), CD8 (cyan), CD4 (yellow), FOXP3 (orange), and GZMB (green) in H22 SLC1A5‐KD tumor tissues.

To corroborate these findings, we performed multiplex immunofluorescence staining of tumor sections. Consistent with the flow cytometry results, *Slc1a5* knockdown markedly reduced CD4^+^FOXP3^+^ Treg‐cell abundance while increasing CD8^+^GZMB^+^ CTL infiltration within the TME (Figure 6D,E).

Collectively, these results demonstrate that *Slc1a5* knockdown reduces immunosuppressive Treg‐cell infiltration and concomitantly enriches CTLs within the tumor immune microenvironment. This shift remodels the immunological landscape from an immune‐cold phenotype toward an immune‐hot phenotype.

### 2.6 ^18^F‐FGln Micro‐PET/CT Imaging Monitors Dynamic Changes in Gln Uptake After V‐9302 Treatment to Assess Tumor Response to Immunotherapy in HCC

Based on our preliminary findings, we confirmed that Gln uptake in HCC cells suppresses antitumor immunity, suggesting that this pathway may represent a therapeutic target in HCC. V‐9302, an inhibitor of SLC1A5/SNAT2, effectively blocks Gln uptake in tumor cells while sparing T cells [24]. Moreover, ^18^F‐FGln PET/CT imaging reflects Gln uptake in HCC and enables evaluation of responses to antitumor immunotherapy. These findings prompted us to investigate whether V‐9302‐mediated inhibition of tumor Gln uptake could enhance antitumor immunotherapy in HCC and whether ^18^F‐FGln PET/CT could be used to evaluate responses to V‐9302 combined with immunotherapy.

To address these questions, H22 tumor‐bearing mice underwent baseline ^18^F‐FGln micro‐PET/CT imaging, followed by V‐9302 administration and repeat ^18^F‐FGln micro‐PET/CT imaging (Figure 7A). Imaging showed a marked reduction in tumoral ^18^F‐FGln uptake after V‐9302 treatment (Figure 7B). Quantitative analysis showed that tumoral ^18^F‐FGln uptake decreased from 14.60 ± 1.81%ID/g before treatment to 7.23 ± 1.30%ID/g after V‐9302 treatment (*p* < 0.05; Figure 7C), and the T/NT ratio decreased from 3.38 ± 0.24 to 1.46 ± 0.09 (*p* < 0.05; Figure 7D). These results indicate that V‐9302 significantly reduces Gln uptake in H22 tumors.

**FIGURE 7:**
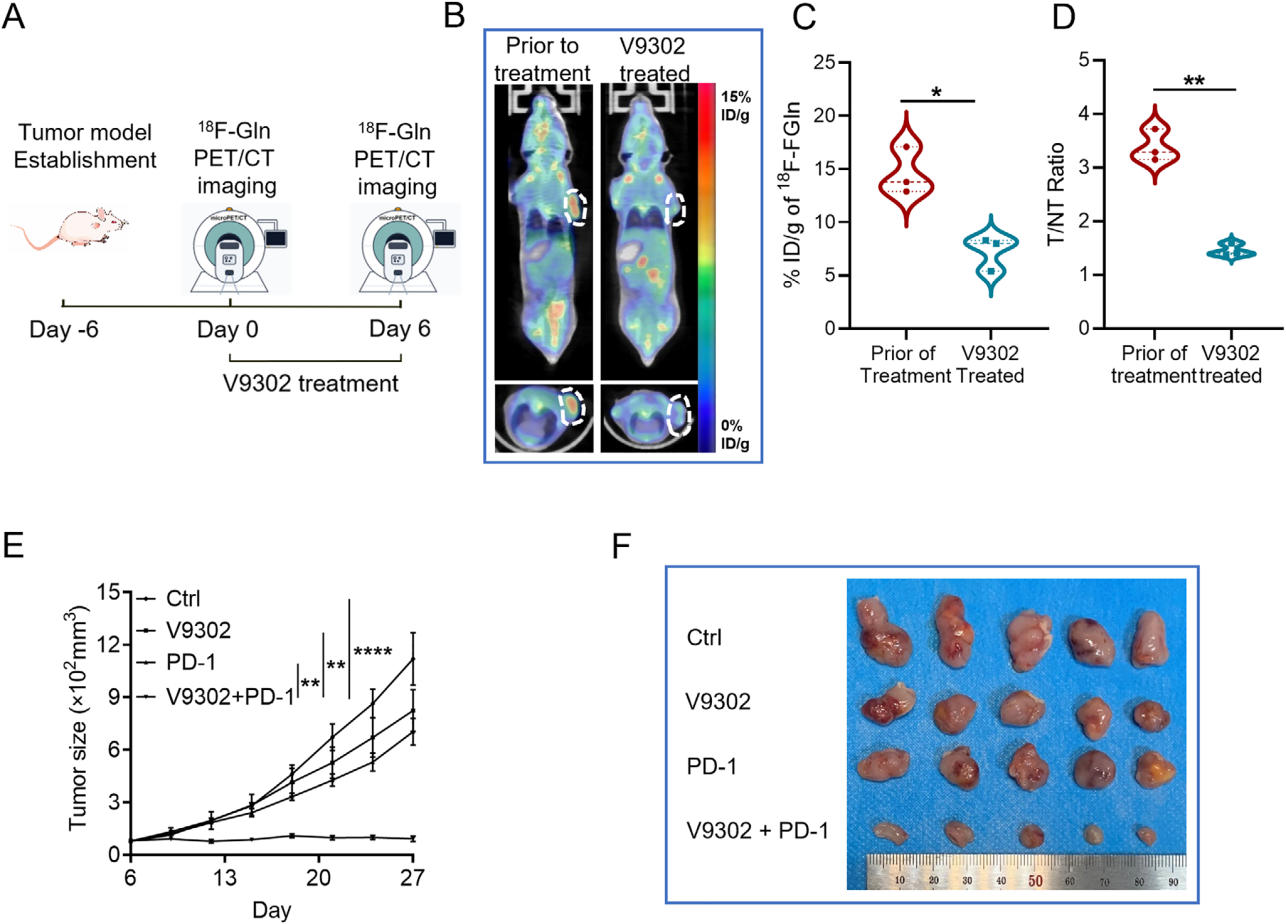
^18^F‐FGln micro‐PET/CT monitors V‐9302‐induced changes in Gln uptake and predicts immunotherapy response in HCC. (A) Schematic overview of the PET imaging and treatment strategy in H22 tumor‐bearing mice. (B) Representative ^18^F‐FGln micro‐PET/CT images of H22 tumor models before and after V‐9302 treatment. (C) Quantification of tumoral ^18^F‐FGln uptake in H22 tumor models before and after V‐9302 treatment (n = 3; **p = 0.0094). (D) Tumor‐to‐normal tissue (T/NT) ratios derived from ^18^F‐FGln micro‐PET/CT images of H22 tumor models before and after V‐9302 treatment (n = 3; ***p = 0.0005). (E) Tumor growth curves of H22 tumors after treatment with anti‐mouse PD‐1 antibody (10 mg/kg), V‐9302 (50 mg/kg), or saline (n = 5). (F) Representative photographs of H22 tumors after treatment with anti‐mouse PD‐1 antibody (10 mg/kg), V‐9302 (50 mg/kg), or saline.

We further evaluated the therapeutic efficacy of V‐9302, anti‐PD‐1 therapy, and their combination in HCC (Figure S6A). In the H22 tumor model, combination therapy with V‐9302 and anti‐PD‐1 markedly suppressed tumor growth compared with control treatment and showed greater efficacy than either monotherapy (Figure 7E,F and Figure S6B). The tumor inhibition rate was 26.36% for V‐9302 alone, 37.15% for PD‐1 alone, and 91.71% for the combination of V‐9302 and PD‐1. Body weight monitoring showed no significant differences between the combination therapy and control groups (Figure S6C).

In summary, V‐9302 significantly reduced tumoral ^18^F‐FGln uptake in the H22 HCC model. Furthermore, V‐9302 substantially potentiated the antitumor efficacy of anti‐PD‐1 therapy, highlighting its potential as an adjunctive strategy for HCC treatment. These findings underscore the ability of ^18^F‐FGln micro‐PET/CT to dynamically monitor V‐9302‐mediated changes in tumor Gln uptake, providing a basis for optimizing immunotherapy decisions in patients with HCC.

### 2.7 ^18^F‐FGln Micro‐PET/CT Imaging Monitors Gln Uptake and Predicts Immunotherapy Response in HCC Organoid‐T‐Cell Co‐Culture Systems

Given that fewer than 10% of cancer therapies successfully translate from preclinical animal models to patients [25], we established a patient‐derived organoid (PDO)‐T‐cell co‐culture system. This platform was designed to further validate the utility of ^18^F‐FGln PET/CT imaging for assessing Gln uptake in HCC tissues and to explore its potential for guiding immunotherapy management (Figure S7A). IHC was first used to identify HCC organoids with low or high SLC1A5 expression. Organoids derived from Patients 1, 2, and 3 exhibited low SLC1A5 expression, whereas those derived from Patients 4, 5, and 6 showed high SLC1A5 expression (Figure S7B,C). Organoids from Patients 1 and 4 were subsequently used to establish patient‐derived organoid xenografts (PDOX) for ^18^F‐FGln micro‐PET/CT imaging. Patient 1‐derived PDOX, which had low SLC1A5 expression, showed low ^18^F‐FGln uptake, whereas Patient 4‐derived PDOX, which had high SLC1A5 expression, showed high ^18^F‐FGln uptake (Figure 8A). Quantitative analysis showed significantly higher ^18^F‐FGln uptake in Patient 4‐derived PDOX than in Patient 1‐derived PDOX (26.90 ± 1.22%ID/g vs. 14.43 ± 1.60%ID/g, *p* < 0.05; Figure 8B). The T/NT ratios were also higher in Patient 4‐derived PDOX than in Patient 1‐derived PDOX (18.52 ± 4.56 vs. 7.62 ± 1.58, *p* < 0.05; Figure 8C).

**FIGURE 8:**
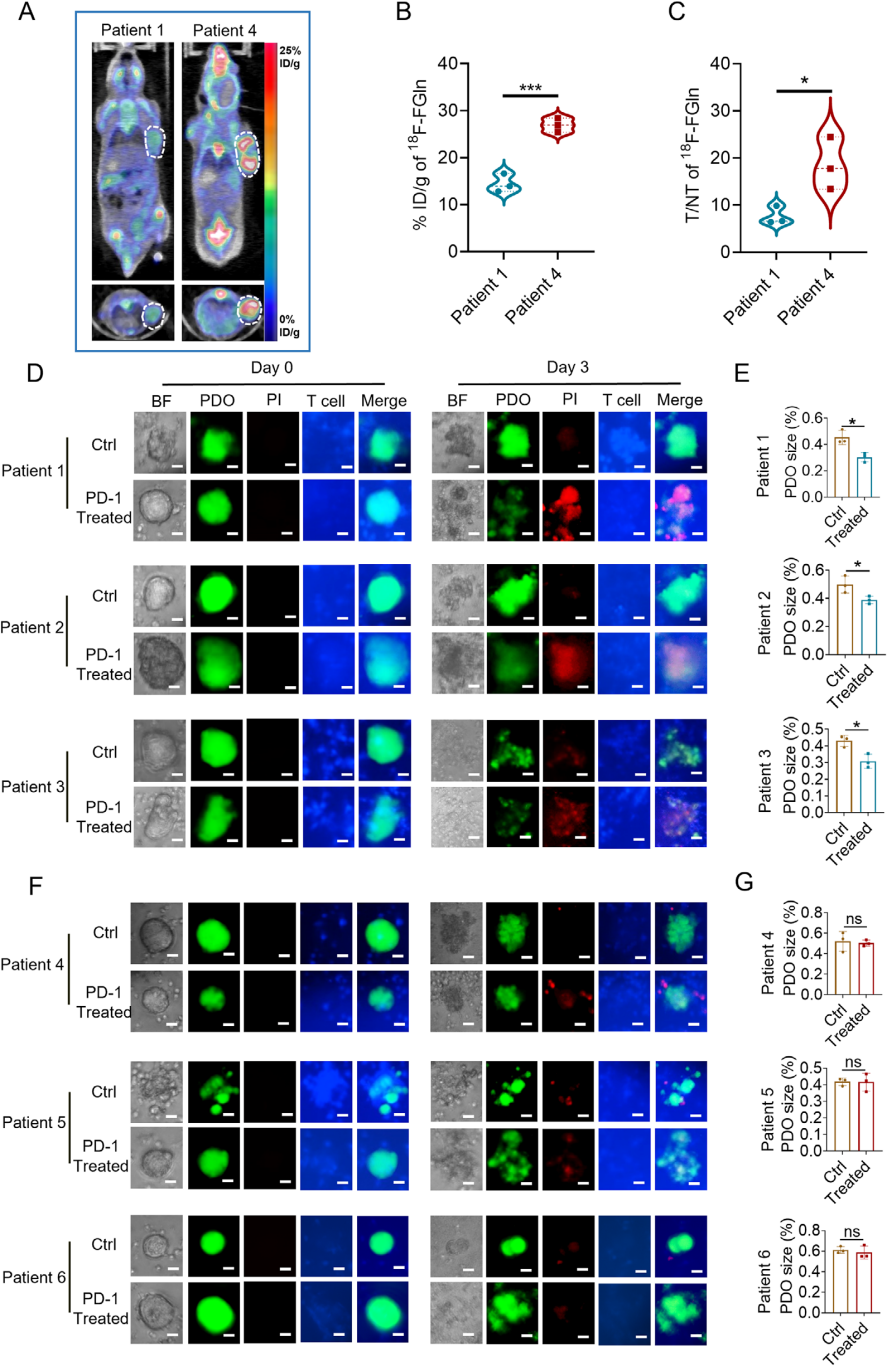
^18^F‐FGln micro‐PET/CT predicts immunotherapy response through metabolic monitoring in HCC organoid‐T‐cell co‐cultures. (A) Representative ^18^F‐FGln micro‐PET/CT images of patient‐derived organoid xenografts (PDOX) with low or high SLC1A5 expression. White dashed circles indicate tumor locations. (B) Quantification of tumoral ^18^F‐FGln uptake in PDOX models with low or high SLC1A5 expression (n = 3; ***p = 0.0009). (C) Tumor‐to‐normal tissue (T/NT) ratios derived from ^18^F‐FGln micro‐PET/CT images of PDOX models with low or high SLC1A5 expression (n = 3; *p = 0.033). (D) Representative bright‐field (BF) and immunofluorescence images of PDOs, PI, T cells, and merged channels in control and anti‐PD‐1‐treated HCC PDOs with low SLC1A5 expression. Scale bars: 20 µm. (E) Quantification of growth in low‐SLC1A5 HCC PDOs in control and anti‐PD‐1‐treated groups at 3 days after co‐culture (n = 3; *p < 0.05). (F) Representative bright‐field (BF) and immunofluorescence images of PDOs, PI, T cells, and merged channels in control and anti‐PD‐1‐treated HCC PDOs with high SLC1A5 expression. Scale bars: 20 µm. (G) Quantification of growth in high‐SLC1A5 HCC PDOs in control and anti‐PD‐1‐treated groups at 3 days after co‐culture (n = 3; *p < 0.05).

To evaluate the inhibitory effect of PD‐1 blockade on organoid growth, we established organoid‐T‐cell co‐culture systems. As shown in Figure 8D, compared with the control group, PD‐1 blockade induced a marked increase in PI‐positive staining and a corresponding reduction in Green CMFDA staining in organoids derived from Patients 1, 2, and 3, indicating increased cell death and reduced organoid viability. Consistent with these findings, PD‐1 blockade substantially suppressed the growth of organoids from Patients 1, 2, and 3 (Figure 8E). In contrast, organoids derived from Patients 4, 5, and 6, which exhibited high SLC1A5 expression, showed little response to PD‐1 blockade. In these cultures, PD‐1 treatment did not cause any appreciable increase in PI staining or decrease in Green CMFDA staining, and organoid viability remained largely unchanged relative to the control group (Figure 8F). Accordingly, PD‐1 blockade failed to significantly inhibit growth in the same co‐culture systems (Figure 8G).

Collectively, these results demonstrate that organoids with low SLC1A5 expression are susceptible to PD‐1 blockade‐induced growth suppression in organoid‐T‐cell co‐culture systems, whereas those with high SLC1A5 expression are refractory to treatment. Furthermore, ^18^F‐FGln micro‐PET/CT imaging may enable prediction of organoid responsiveness to PD‐1 blockade.

Overall, these data confirm that ^18^F‐FGln PET/CT provides a reliable noninvasive measure of Gln uptake in HCC. Tumors with elevated Gln uptake were refractory to immunotherapy, whereas those with lower uptake were more susceptible. In addition, V‐9302 effectively reduced Gln uptake in HCC and reversed immunotherapy resistance, with this metabolic decline dynamically captured by ^18^F‐FGln PET/CT. Together, these findings support the potential of ^18^F‐FGln PET/CT for clinical translation in predicting immunotherapeutic outcomes and guiding optimized immunotherapy strategies in HCC.

## 3 Discussion

HCC remains a major global health challenge, with increasing incidence and mortality [26, 27]. It frequently presents at an advanced stage, and recurrence after surgical treatment is common, making systemic therapy an essential component of management [28]. The combination of atezolizumab, an anti‐PD‐L1 antibody, and bevacizumab, an anti‐VEGFA antibody, has shown superior efficacy to sorafenib in advanced HCC and established a new first‐line benchmark, with a median OS of 19 months [29]. Although ICIs have transformed cancer treatment, confirmatory phase III trials of nivolumab (CheckMate 459) and pembrolizumab (KEYNOTE‐240) failed to meet prespecified statistical significance thresholds, despite ORRs of 14–17% and response durations exceeding 12 months [30, 31]. Their clinical impact is further limited by toxicity, with TRAEs reported in approximately 20–60% of patients receiving anti‐PD‐1 monotherapy and severe irAEs in 10–20% of patients receiving anti‐PD‐1/PD‐L1 antibodies and in about 25% receiving anti‐CTLA‐4 antibodies [32, 33]. Because immunotherapy does not benefit all patients, identifying intrinsic resistance would facilitate alternative treatment selection and reduce unnecessary costs and resource use. These challenges underscore the need for biomarkers that can more accurately predict treatment response.

Efforts to identify predictive biomarkers for ICI monotherapy have included PD‐L1 expression, tumor mutational burden, microsatellite instability, and mismatch repair status [34, 35, 36, 37]. However, no single biomarker has demonstrated sufficient predictive performance for immunotherapy response. Moreover, biopsy‐based assessment is invasive and is limited by temporal and spatial heterogeneity within primary and metastatic lesions. In the context of precision medicine, there is a growing need for a noninvasive, accurate, and reproducible approach to monitor therapeutic response during immunotherapy. Although ^18^F‐FDG PET/CT has been explored for early response assessment [38], its uptake correlates poorly with treatment outcomes [39]. In contrast, immune‐targeted radiotracers, including ^89^Zr‐pembrolizumab [40], ^89^Zr‐atezolizumab [41], and ^89^ZrDf‐IAB22M2C [42], offer molecular specificity, but current probes remain constrained by heterogeneous target expression and nonspecific uptake in the liver, bones, spleen, lymph nodes, and bone marrow.

Cancer‐cell dependence on Gln reduces local Gln availability, thereby impairing antitumor T‐cell function and facilitating immune evasion. Recent studies have shown that Gln metabolic reprogramming in cancer cells contributes to immune regulation and tumor drug resistance. Upregulated SLC1A5 expression in tumor cells limits Gln availability to immune cells and suppresses antitumor immune function [5]. By selectively inhibiting tumor‐cell Gln uptake with V‐9302, Gln availability in the TME increases, thereby enhancing CD8^+^ T‐cell‐mediated antitumor responses [43]. Previous studies have established Gln as a critical metabolic checkpoint that orchestrates the crosstalk between tumor cells and conventional type 1 dendritic cells (cDC1s), thereby licensing cDC1 function in priming CD8^+^ T cell responses [11]. There is accumulating evidence indicates that altered Gln metabolism is a common characteristic among different types of cancers, such as colon, renal and melanoma cancers, highlighting its potential as a therapeutic target [14, 44]. Several compounds targeting Gln intake or enzymes involved in Gln catabolism are currently being evaluated in preclinical and early‐stage clinical trials [45]. Furthermore, Gln metabolism is emerging as a promising target for monitoring immune responses and guiding immunotherapy strategies in tumors. Quantifying Gln in vivo using translational imaging techniques may help identify Gln as a biomarker, stratify patients for novel therapies, especially immunotherapies, and accelerate the translation of emerging treatments into the clinic [46].

^18^F‐FGln was first synthesized by Kung and colleagues in 2010 and subsequently validated as a radiotracer for oncologic imaging [47]. Emerging evidence suggests that it can capture the heterogeneous Gln metabolic landscape of tumors, including cerebral metastases, and support integrated diagnostic and therapeutic monitoring [48]. In addition, Manning and colleagues showed that ^18^F‐FGln PET/CT provides a quantitative, noninvasive measure of SLC1A5 expression in preclinical non‐small cell lung cancer (NSCLC) models [49]. Together, these advances support ^18^F‐FGln PET as a promising noninvasive modality for evaluating Gln uptake and optimizing Gln‐targeted interventions in cancer [50]. Given the emerging link between tumor Gln uptake and antitumor immune responses, we hypothesized that Gln PET imaging could identify patients most likely to benefit from immunotherapy and help guide individualized treatment.

The principal findings of this study are as follows. Analysis of the TCGA‐LIHC dataset showed that *SLC1A5* expression was inversely associated with patient prognosis (Figure 1A) and positively associated with histological grade (Figure 1B). HCC tumors with high *SLC1A5* expression exhibited an immunologically cold TME (Figure 1C–J). After selecting H22 cells, which showed high ^18^F‐FGln uptake, and Hepa1‐6 cells, which showed low ^18^F‐FGln uptake, we established subcutaneous tumor models and assessed their response to anti‐PD‐1 therapy. H22 tumors showed high ^18^F‐FGln uptake (maximum tumor uptake = 26.30 ± 2.30%ID/g; Figure 2A–D) and high SLC1A5 expression (Figure 2H,I), but limited sensitivity to immunotherapy (Figure 2K), whereas Hepa1‐6 tumors showed low ^18^F‐FGln uptake (maximum tumor uptake = 6.87 (1.9) ± 0.21%ID/g, Figures 2A–D), low SLC1A5 expression (Figure 2H,J), and effective control by PD‐1 blockade (Figure 2L). After generating *Slc1a5*‐KD H22 cells, we observed that *Slc1a5* knockdown suppressed tumor growth after PD‐1 therapy (Figure 3D,E) and reduced ^18^F‐FGln uptake (Figure 3F–I). Transcriptomic profiling further showed upregulation of cytotoxic T‐cell effector genes, including *Cd8a*, *Cd8b1*, *Gzmb*, *Gzmf*, and *Prf1* (Figure 4A,B), together with enrichment of pathways associated with antitumor immunity (Figure 4C–E). Single‐cell RNA‐seq, flow cytometry, and multiplex immunofluorescence consistently demonstrated reduced Treg abundance and increased CTL infiltration after *Slc1a5* knockdown. To overcome resistance in high Gln uptake HCC, we combined V‐9302 with anti‐PD‐1 therapy, and ^18^F‐FGln PET/CT dynamically tracked the resulting metabolic changes (Figure 7A–D). To more closely recapitulate the human setting, we established a co‐culture system comprising T cells and patient‐derived HCC organoids. We selected organoids with high and low SLC1A5 expression, and utilized ^18^F‐FGln micro‐PET/CT to monitor Gln uptake (Figure 8A–C). In the patient‐derived organoid‐T‐cell co‐culture system, high‐SLC1A5 organoids showed only modest sensitivity to PD‐1 blockade, whereas low‐SLC1A5 organoids were strongly suppressed, reinforcing the relevance of Gln metabolic reprogramming in immunotherapy responsiveness (Figure 8D–G).

Despite the consistent findings presented above, several limitations warrant consideration. First, the in vivo experiments were performed in subcutaneous models, which do not fully recapitulate the architecture, perfusion, or immune context of spontaneous human HCC. Whether these observations are reproducible in the orthotopic liver microenvironment, where immune trafficking and metabolic crosstalk differ substantially, remains to be established. Second, bulk RNA‐seq and single‐cell RNA‐seq lack spatial resolution, limiting our ability to define topological interactions between SLC1A5‐expressing tumor cells and adjacent immune cells. Future spatial transcriptomics or imaging mass cytometry studies would be valuable. Third, the organoid‐T‐cell co‐culture system used established organoid lines without autologous immune matching and was based on a limited sample size, which may restrict generalizability across HCC subtypes. Finally, our mechanistic analysis was limited to transcriptomic data and lacked proteomic or metabolomic validation, leaving unresolved whether the immune‐related changes were direct consequences of SLC1A5 loss or indirect effects of downstream metabolic reprogramming. These limitations do not undermine our main conclusions, but they do highlight the need for future studies using orthotopic models, spatial profiling, and deeper mechanistic dissection of metabolic‐immune crosstalk in HCC.

In summary, our study identifies Gln uptake as a checkpoint that promotes immune evasion in HCC. High Gln uptake, reflected by high ^18^F‐FGln uptake, is associated with poor prognosis and PD‐1 resistance, whereas low uptake is associated with enhanced cytotoxic T‐cell activity and reduced Treg infiltration. V‐9302 sensitizes refractory HCC to anti‐PD‐1 therapy. Thus, Gln uptake functions as both a biomarker and a therapeutic target, warranting orthotopic validation and mechanistic dissection before clinical translation.

## 4 Materials and Methods

### 4.1 TCGA Data Analyses

TCGA‐LIHC patients (n = 368) were stratified into high‐ and low‐*SLC1A5* expression groups according to the median *SLC1A5* expression level. Kaplan‐Meier survival curves were generated, and the log‐rank test was used to compare overall survival between groups. Patients were further classified into pathological grades G1‐G4, and *SLC1A5* expression among groups was compared using the Mann‐Whitney U test. Spearman correlation analysis was performed to assess the association between *SLC1A5* expression and immune checkpoint molecules. All statistical analyses and figures were generated using R software (version 4.5.3). A two‐sided *p*‐value < 0.05 was considered statistically significant.

### 4.2 Cell Cultures

Murine HCC cell lines, including RH‐35, Hepa1‐6, CBRH‐7919, Hepa1c1c7, and H22, were obtained from the Cell Bank of the Shanghai Academy of Chinese Sciences. RH‐35 and Hepa1‐6 cells were cultured in Dulbecco's modified Eagle's medium (DMEM; Basalmedia, Shanghai, China). CBRH‐7919 and H22 cells were cultured in RPMI‐1640 medium (Basalmedia, Shanghai, China) supplemented with 10% fetal bovine serum (FBS; HyClone) and 1% penicillin‐streptomycin (Invitrogen). Hepa1c1c7 cells were cultured in Minimum Essential Medium Alpha (α‐MEM) without nucleosides, supplemented with 2 mM L‐glutamine and 10% FBS.

### 4.3 Radiosynthesis of ^18^F‐FDG and ^18^F‐FGln

^18^F‐FDG was automatically produced using an Explora FDG4 module and a cyclotron (CTI RDS Eclipse ST; Siemens, Knoxville, TN, USA), as described previously [51]. ^18^F‐FGln was radiosynthesized from a protected tosylate precursor according to a previously reported procedure with minor modifications [24]. The final ^18^F‐FGln product was obtained in approximately 5% non‐decay‐corrected radiochemical yield and had a radiochemical purity of 99%, as determined by chiral HPLC (Figure S8).

### 4.4 Cell Uptake

RH‐35, Hepa1‐6, CBRH‐7919, Hepa1c1c7, and H22 cells were seeded in 24‐well plates at a density of 1 × 10^5 cells per well and incubated overnight. Cells were then incubated with ^18^F‐FGln (370 kBq/well) at 37°C for 60 min. After incubation, cells were washed three times with ice‐cold PBS and harvested using 0.25% trypsin/0.02% EDTA (HyClone). Cell suspensions were collected, and radioactivity was measured using a gamma counter (SN‐6110; Rihuan Optoelectronic Instruments, Shanghai Institute of Nuclear Research, Shanghai, China). Cell uptake was expressed as the percentage of total input radioactivity after decay correction. Experiments were performed in triplicate wells. The cell uptake assay methods for H22 WT and H22 KD were the same as above.

### 4.5 Animal Models

All animal procedures were performed in accordance with protocols approved by the Institutional Animal Care and Use Committee of Fudan University. Hepa1‐6 cells (2 × 10^5) or H22 cells (2 × 10^5) suspended in 100 µL phosphate‐buffered saline (PBS) were subcutaneously injected into the right shoulder region of female wild‐type C57BL/6 or BALB/c mice, respectively. The mice used were approximately 4 weeks of age and weighed approximately 20 g. In the tumor inhibition experiments, mice bearing tumors were euthanized when the mean tumor volume of the tumor‐bearing mouse model exceeded 1200 mm^3^ or the body weight of the mouse fell below 16 g. PD‐1 antibody (A2122, Selleck Chemicals) was administered intraperitoneally at 10 mg/kg twice per week. The SLC1A5 inhibitor V‐9302 (HY‐112683, MedChemExpress) was administered intraperitoneally at 50 mg/kg daily for 5 days [9]. Tumor volumes were measured using the modified ellipsoidal formula V = (length × width^2^)/2 every 2 days. Tumor inhibition rate (%) = (average tumor volume of the control group—average tumor volume of the experimental group) / average tumor volume of the control group × 100%.

### 4.6 Micro‐PET/CT Scanning and Biodistribution Procedure

Approximately 7.4 MBq of ^18^F‐FDG or ^18^F‐FGln was administered intravenously to mice under isoflurane anesthesia (n = 3/group). Static PET images were acquired 1 h after injection using a 10‐min acquisition protocol on an Inveon micro‐PET/CT scanner (Siemens). CT images were acquired immediately before PET scanning to provide anatomical reference data for PET image co‐registration. PET images were reconstructed using a three‐dimensional ordered‐subsets expectation maximization algorithm and co‐registered with CT images using Inveon Research Workplace software (version 4.0; Siemens). For each scan, three‐dimensional regions of interest (3D ROIs) were manually drawn over tumors and normal tissues. Tracer accumulation in tumors and muscle was calculated from maximum values within multiple ROIs and expressed as the percentage injected dose per gram of tissue (%ID/g). For the T/NT calculation, the radioactivity uptake value of the muscle at the left shoulder of the mouse was used as the radioactivity uptake value of the normal tissue. After ^18^F‐FGln micro‐PET/CT imaging, tumor‐bearing mice were euthanized, and tumors and selected tissues or organs were harvested, weighed, and counted using a gamma counter. Biodistribution results were expressed as %ID/g.

### 4.7 In Vitro Autoradiography and Hematoxylin and Eosin Staining

After imaging, tumor‐bearing mice were euthanized, and tumor tissues were collected (n = 3 per group). Tumors were bisected, with one half fixed in paraformaldehyde for hematoxylin and eosin (H&E) staining and the other half placed on a phosphor screen for autoradiography. Images were acquired by scanning the phosphor screen using a Typhoon 2.0 scanner (GE Healthcare).

### 4.8 Histology and Immunohistochemistry Staining

Tissues were harvested and fixed in 4% paraformaldehyde. Immunohistochemical staining was performed using an anti‐ASCT2 antibody (rabbit, reactive with human and mouse; 1:100; A23156, ABclonal) overnight at 4°C. After incubation with the primary antibody, positive cells were visualized using DAB+ as the chromogen. IHC images were acquired using the Dako Autostainer Link 48 system (Agilent). Three random fields per slide were selected for quantitative analysis. The IHC score ranged from 0 to 8 and was calculated as the sum of the staining intensity score and the percentage of positive cells score. Staining intensity was scored on a scale of 0 to 3: 0, negative; 1, weak; 2, moderate; and 3, strong. The percentage of positive cells was scored on a scale of 0 to 5: 0, no staining; 1, 1% to 10%; 2, 11% to 25%; 3, 26% to 50%; 4, 51% to 75%; and 5, 76% to 100%.

### 4.9 *Slc1a5* Knockdown

*Slc1a5* knockdown was performed in mouse H22 cells using the psi‐LVRU6P vector to express either target‐specific shRNA or control shRNA. ShRNA sequences were cloned into the BamHI and EcoRI restriction sites of the psi‐LVRU6P vector. The following oligonucleotide sequences were used for shRNA‐mediated knockdown: control shRNA, GCTTCGCGCCGTAGTCTTA; mouse *Slc1a5* shRNA, GGAATCTGTCATGTGAATATC.

### 4.10 RNA Isolation, Library Preparation, Sequencing and Analysis

Total RNA was extracted from samples using TRIzol reagent (Invitrogen, CA, USA) according to the manufacturer's instructions. RNA purity and concentration were assessed using a NanoDrop 2000 spectrophotometer (Thermo Scientific, USA), and RNA integrity was evaluated using an Agilent 2100 Bioanalyzer (Agilent Technologies, CA, USA). RNA‐seq libraries were prepared using the VAHTS Universal V10 RNA‐seq Library Prep Kit (Premixed Version). Libraries were sequenced on an Illumina NovaSeq 6000 platform to generate 150‐bp paired‐end reads.

Raw FASTQ reads were processed using fastp to remove low‐quality sequences, yielding clean reads. Gene expression quantification was performed using HTSeq‐count, and FPKM values were subsequently calculated. Sample reproducibility was assessed by principal component analysis (PCA) in R (version 3.2.0).

Differentially expressed genes (DEGs) were identified using DESeq2, with thresholds set at |fold change| > 2 and an adjusted *p*‐value (q) < 0.05. Hierarchical clustering was used to visualize expression patterns across groups, and radar plots of the top 30 DEGs were generated using the R package ggradar to display upregulated and downregulated profiles.

Functional enrichment analyses, including Gene Ontology (GO), KEGG, Reactome, and WikiPathways, were conducted in R based on a hypergeometric distribution. Significant terms were visualized using column, chord, and bubble plots. Gene set enrichment analysis (GSEA) was performed using GSEA software, with genes ranked by differential expression signal to test for enrichment of predefined gene sets at the extremes of the ranked list.

### 4.11 QRT‐PCR

Total RNA was extracted using FreeZol Reagent (R711‐01, Vazyme) and reverse transcribed into cDNA using HiScript III RT SuperMix for qPCR (+gDNA wiper) (R323‐01, Vazyme). Quantitative PCR was performed on a 7300 Plus Real‐Time PCR System (7300Plus, Applied Biosystems) using Taq Pro Universal SYBR qPCR Master Mix (Q712, Vazyme) and the primers listed in Table S1. The thermal cycling program consisted of 95°C for 10 min, followed by 40 cycles of 95°C for 15 s and 60°C for 1 min. Relative gene expression was calculated using the 2^‐ΔΔCt method and normalized to ACTB. All reactions were performed in triplicate.

### 4.12 Single‐cell RNA Sequencing

Fresh tissue specimens were placed in MACS Tissue Storage Solution (Miltenyi Biotec) immediately after resection. Samples were minced and digested in RPMI‐1640 containing 10% FBS, Tumor Dissociation Kit (Miltenyi), 30 U/mL DNase I, and 1 mg/mL neutral protease II for 40 min at 37°C. The resulting cell suspension was filtered through a 70‐µm strainer, centrifuged at 300 g for 5 min, treated with red blood cell lysis buffer, washed with PBS containing 0.04% BSA, re‐filtered through a 40‐µm strainer, and stained with Calcein‐AM and Draq7 for viability assessment. Single‐cell libraries were generated using the BD Rhapsody system through microwell‐based cell capture and barcoded bead pairing. After cell lysis, mRNA was captured, reverse transcribed with UMI and cell‐barcode tagging, and subjected to whole‐transcriptome amplification (WTA). Libraries were quantified using a Bioanalyzer 2200 and Qubit and sequenced on a DNBSEQ‐T7 platform to generate 150‐bp paired‐end reads.

Raw reads were trimmed and filtered using fastp, and the resulting reads were aligned to the mm10 reference genome (Ensembl release 100) with STARsolo (v2.7.11b). Cells with more than 200 detected genes and a mitochondrial UMI fraction below 20% were retained. Data were normalized and scaled using Seurat (v4.1.1). PCA based on the top 2,000 highly variable genes and the top 10 principal components was used for t‐SNE/UMAP visualization. Unsupervised clustering was performed at a resolution of 0.8, and marker genes were identified using the Wilcoxon rank‐sum test with log2FC > 0.25, *p* < 0.05, and min.pct > 0.1.

Pathway and gene set activity were evaluated using QuSAGE (v2.16.1), AUCell (v1.18.1), and ssGSEA implemented in GSVA (v1.32.0). Transcription factor networks were inferred using pySCENIC (v0.11.2). Pseudotime and trajectory analyses were performed using Monocle2/3, PAGA (v1.3.3), CytoTRACE (v0.3.3), and CytoTRACE 2. Cell‐cell communication was analyzed using CellPhoneDB and CellChat (v2). Differentially expressed genes were identified using FindMarkers with the same criteria described above. GO and KEGG enrichment analyses were performed using Fisher's exact test with FDR correction.

### 4.13 Multicolor Immunofluorescence

Sections were baked at 65°C for 2 h, deparaffinized, rehydrated, subjected to antigen retrieval, and blocked for endogenous peroxidase activity. Sequential multiplex staining was performed using an anti‐SLC1A5 primary antibody (A23156, Abclone, 1:500) overnight at 4°C, followed by an HRP‐polymer anti‐rabbit secondary antibody for 30 min at room temperature and TYR‐570 development for 3–10 min, with PBST washing after each step. Antigen retrieval and blocking were then repeated, followed by sequential staining for CD8 (AFRM0004, AiFang Biological, 1:1000), CD4 (AFRM0003, AiFang Biological, 1:1000), Granzyme B (AFRM0352, AiFang Biological, 1:1400), and FOXP3 (AFRM0359, AiFang Biological, 1:400), using the same secondary antibody and the corresponding TSA fluorescent dyes (TYR‐480, TYR‐690, TYR‐520, and TYR‐620, respectively), with PBST washing after each round. Nuclei were counterstained with DAPI, and the sections were mounted and imaged under a fluorescence microscope.

### 4.14 Flow Cytometry

Tumors were collected, mechanically minced, and digested in buffer containing DNase I (50 µg/mL; MilliporeSigma), collagenase (2 mg/mL; MilliporeSigma), DMEM, FBS, and penicillin‐streptomycin for 30 min at 37°C. The digested tissue was filtered through 70‐µm strainers (BD Falcon) and washed with FACS buffer consisting of PBS supplemented with 0.5% endotoxin‐free FBS, 2 mM EDTA, and 25 mM HEPES. Cells were collected and analyzed by flow cytometry. Tumor‐derived cells were blocked with anti‐CD16/32 antibody (1 µg per 10^6 cells in 100 µL dilution buffer; catalog 101319; BioLegend) and stained with the indicated surface antibodies. Dead cells were identified using Fixable Viability Dye 545 (catalog S0D0014, STARTER). Fluorochrome‐conjugated or biotinylated antibodies, together with their dilutions and manufacturers, were as follows: APC‐Cy7 anti‐mouse CD45 (1:200; catalog 561037, BD Pharmingen), Alexa Fluor 700 hamster anti‐mouse CD3e (1:200; catalog 557984, BD Pharmingen), PerCP‐Cy5.5 anti‐mouse CD4 (1:200; catalog 550954, BD Pharmingen), FITC anti‐mouse CD8 (1:200; catalog S0B5120, STARTER), BV605 anti‐mouse CD25 (1:200; catalog 563061, BD Pharmingen), PE‐Cy7 anti‐mouse FOXP3 (1:200; catalog 25‐5773‐82, Thermo), PE anti‐mouse GZMB (1:200; catalog 12‐8898‐82, Thermo), APC anti‐mouse Perforin (1:200; catalog 17‐9392‐80, Thermo), BV421 anti‐mouse CD44 (1:200; catalog 563970, BD Pharmingen), PE‐CF594 anti‐mouse CD62L (1:200; catalog 562404, BD Pharmingen), and BV650 anti‐mouse PD‐1 (1:200; catalog 744546, BD Pharmingen). Intracellular staining was performed after fixation and permeabilization (catalog 562574, BD Pharmingen) according to the manufacturer's instructions. Data were acquired using a Beckman Coulter CytoFLEX flow cytometer and analyzed with FlowJo software (version 10.8.1; Tree Star). The reported percentages were calculated based on the respective T‐cell subsets. The gating strategy was present in Figure S5.

### 4.15 Organoid–T Cell Co‐Cultures

Human HCC organoids (D1 Medical Technology (Shanghai) Co., Ltd.) were recovered from cryopreservation and cultured in NGC Organoid medium (DIMed) at 37°C in 5% CO2, with medium changed every 3 days. When more than 70% of organoids reached a diameter of 50–100 µm, Matrigel was removed, and organoids were stained with 12.5 µM Green CMFDA for 1.5 h, followed by washing with PBS. A 96‐well plate was pre‐coated with 20 µL of 50% Matrigel, after which 250 organoids per well, suspended in 10% Matrigel, were seeded and overlaid with 100 µL medium.

PBMC‐derived T cells were activated in X‐VIVO 15 medium supplemented with 200 IU/mL IL‐2 for 3–6 days until clustering was observed. Cells with viability of at least 89% were stained with 12.5 µm CellTracker Blue CMAC for 2 h, resuspended in medium containing 200 IU/mL IL‐2 and 200 ng/mL PI, and added to organoid wells at 2 × 10^5 cells per well. The experimental groups were Blank (organoids only), Control (organoids plus T cells), and Treatment (organoids plus T cells plus 12.5 µg/mL anti‐PD‐1).

On days 0 and 3, Z‐stack images were acquired under bright‐field and green/red/blue channels at 3× and 20× magnification. Quantitative analysis of 3× images was performed in ImageJ to calculate organoid viability as the day 3/day 0 fluorescence ratio, normalized to the Blank group. Statistical comparisons between groups were performed using an unpaired t‐test in GraphPad Prism. Data are presented as mean ± SD from at least three independent experiments.

### 4.16 Statistics

Statistical comparisons between two groups were performed using an unpaired two‐tailed Student's t‐test. Tumor growth and mouse body weight curves were analyzed using two‐way repeated‐measures ANOVA followed by multiple comparisons at each time point. The uptake of ^18^F‐FGln by H22 tumors before and after V‐9302 treatment was compared using a paired t‐test. Statistical analyses were performed using GraphPad Prism 9 (GraphPad Software Inc.). A two‐sided *p*‐value < 0.05 was considered statistically significant.

## Author Contributions

**Shaoli Song** and **Fei Liu** designed and guided the project. **Yi Liu**, **Jindian Li** and **Xiaoyu Pan** contributed equally to this work, who designed, carried out, and analyzed most of the experiments. **Jianping Zhang**, **Simin He**, **Xuan Huang** and **Xiaoping Xu** helped perform micro‐PET imaging and data analysis. Yi Liu, Fei Liu and Shaoli Song wrote the manuscript. Shaoli Song and Fei Liu edited the manuscript. All authors read and agreed to the final version of the manuscript.

## Ethics Statement

Mouse studies were approved by the Research Ethics Committee of the FUSCC. All mouse studies were carried out in accordance with the requirements of the Animal Research Committee of Fudan University regarding the care and use of experimental animals in research (FUSCC‐IACUC‐S20210374).

## Conflicts of Interest

The authors declare no conflicts of interest.

## Supporting information




**Supporting File**: advs78622‐sup‐0001‐SuppMat.docx.

## Data Availability

Research data are not shared.
