# Supplementary material for: 18F‐FGln PET/CT Imaging of Glutamine Uptake Has Potential for Predicting Immunotherapy Response in Hepatocellular Carcinoma
Source: Adv Sci (Weinh). 2026 Nov 3:e78622. Online ahead of print. doi: 10.1002/advs.78622 (PMC13731072; doi:10.1002/advs.78622)
Supplement: Supplementary file 1 [file ADVS-9999-e78622-s001.docx]

**Supplementary Materials**

**^18^F-FGln PET/CT Imaging of Glutamine Uptake Has Potential for Predicting Immunotherapy Response in Hepatocellular Carcinoma**

Yi Liu^1, 2, 3, 4, 5, *^, Jindian Li^1, 3, 4, 5 *^, Xiaoyu Pan^1, 3, 4, 5 *^, Xiaoping Xu^1, 3, 4, 5^, Xuan Huang^1, 3, 4, 5^, Simin He^1, 3, 4, 5^, Jianping Zhang^1, 3, 4, 5^, Fei Liu^1, 3, 4, 5^, Shaoli Song^1, 3, 4, 5^

^1^Department of Nuclear Medicine, Fudan University Shanghai Cancer Center, Shanghai 200032, China

^2^Department of Nuclear Medicine, Clinical Oncology School of Fujian Medical University, Fujian Cancer Hospital, Fuzhou, 350014, China

^3^Department of Oncology, Shanghai Medical College, Fudan University; Shanghai 200032, China

^4^Center for Biomedical Imaging, Fudan University; Shanghai 200032, China

^5^Shanghai Engineering Research Center of Molecular Imaging Probes, Shanghai 200032, China

*****These authors contributed equally to this work.

**Correspondence:** Shaoli Song (shaoli-song@163.com) and Fei Liu (liufei0808@126.com)

**Keywords:** ^18^F-FGln PET/CT, glutamine metabolism, hepatocellular carcinoma, immunotherapy

**Figure S1. High ^18^F-FGln uptake is associated with poor response to immunotherapy in HCC.** (A) Cellular uptake assay of ^18^F-FGln in CBRH-7919, H22, Hepa1-6, RH-35, and Hepa1c1c7 tumor cells. %AD, percentage administered dose. Data are presented as mean ± SD (n = 5; *****p* < 0.0001). (B) Biodistribution of ^18^F-FGln in H22 and Hepa1-6 tumor models (n = 3; ****p* = 0.0004). (C) Tumor-to-normal tissue (T/NT) ratios derived from ^18^F-FDG micro-PET/CT images of H22 and Hepa1-6 tumor models (n = 3; ns, *p* = 0.8568). (D) Tumor weights of H22 tumor-bearing mice after treatment with anti-mouse PD-1 antibody (10 mg/kg) or saline (n = 5; ns, P = 0.9055). (E) Body weight curves of H22 tumor-bearing mice after treatment with anti-mouse PD-1 antibody (10 mg/kg) or saline (n = 5; ns, *p* = 0.9414). (F) Tumor weights of Hepa1-6 tumor-bearing mice after treatment with anti-mouse PD-1 antibody (10 mg/kg) or saline (n = 5; *****p* < 0.0001). (G) Body weight curves of Hepa1-6 tumor-bearing mice after treatment with anti-mouse PD-1 antibody (10 mg/kg) or saline (n = 5; ns, *p* = 0.0511).

**Figure S2. *Slc1a5* knockdown reduces ^18^F-FGln uptake and sensitizes HCC to immunotherapy.** (A) H22 WT and H22 KD cell uptake of ^18^F-FGln. (B) Biodistribution of ^18^F-FGln in H22 WT and H22 KD tumor models (n = 3; ****p* = 0.0005).

**Figure S3. HCC-intrinsic *Slc1a5* deficiency triggers antitumor immunity.** (A) Venn diagram showing the overlap between differentially expressed genes in H22 KD versus H22 WT tumors and immune-related genes. The immune-related gene list was obtained from the ImmPort database (https://www.immport.org/shared/genelists). (B) KEGG pathway activation profiles showing significant upregulation of immune-related pathways in the H22 KD group compared with the H22 WT group.

**Figure S4. Single-cell RNA-seq analysis reveals distinct immune landscapes in *Slc1a5*-high and *Slc1a5*-low HCC.** (A) Expression profiles of Treg-associated immunosuppressive genes across CD4^+^ T-cell subsets. (B) Violin plots showing the single-cell expression distribution of these genes within each CD4^+^ T-cell subset.

**Figure S5. Gating strategy for flow cytometry.**

**Figure S6. ^18^F-FGln micro-PET/CT monitors V-9302-induced changes in Gln uptake and predicts immunotherapy response in HCC.** (A) Schematic overview of the treatment strategy in H22 tumor-bearing mice. (B) Tumor weight after treatment with anti-mouse PD-1 antibody (10 mg/kg), V-9302 (50 mg/kg), or saline (n = 5; ns, P = 0.5617). (C) Tumor growth curves of H22 tumor-bearing mice after treatment with anti-mouse PD-1 antibody (10 mg/kg), V-9302 (50 mg/kg), or saline (n = 5; ns, P = 0.5617).

**Figure S7. ^18^F-FGln micro-PET/CT predicts immunotherapy response through metabolic monitoring in HCC organoid-T-cell co-cultures.** (A) Schematic illustration of ^18^F-FGln micro-PET/CT for predicting PD-1–mediated inhibition of organoid growth in organoid–T cell co-cultures. (B) Immunohistochemical (IHC) analysis of SLC1A5 expression in organoids derived from Patients 1, 2, and 3. (C) IHC analysis of SLC1A5 expression in organoids derived from Patients 4, 5, and 6.

**Figure S8.** **Radio-HPLC chromatogram of ¹⁸F-FGln.** Analytical radio-HPLC showed a single dominant radioactive peak for ¹⁸F-FGln at a retention time of 10.5 min, with no appreciable radiochemical impurities. Integration of the radiochromatogram demonstrated a radiochemical purity of >98%, confirming the high purity of the formulated tracer. The final molar activity was estimated to be 48.3 GBq/μmol (1.31 Ci/μmol).

**Supplemental Table 1. Primer sequences used in this study.**
